# Supplementary material for: Role of Peroxisome Proliferator-Activated Receptor α-Dependent Mitochondrial Metabolism in Ovarian Cancer Stem Cells
Source: Int J Mol Sci. 2024 Nov 1;25(21):11760. doi: 10.3390/ijms252111760 (PMC11546303; doi:10.3390/ijms252111760)
Supplement: Supplementary file 1 [file ijms-25-11760-s001.zip › ijms-3257706-supplementary.pdf]

Supplementary material

Role of peroxisome-proliferator-activated receptors α-dependent mitochondrial metabolism in ovarian cancer stem cells

Seoyul Lee, Min Joo Shin, Seong Min Choi, Dae Kyoung Kim, Meegyeon Choi, Jun Se Kim, Dong-Soo Suh, Jae Ho Kim, and Seong-Jang Kim

Supplemental Figures

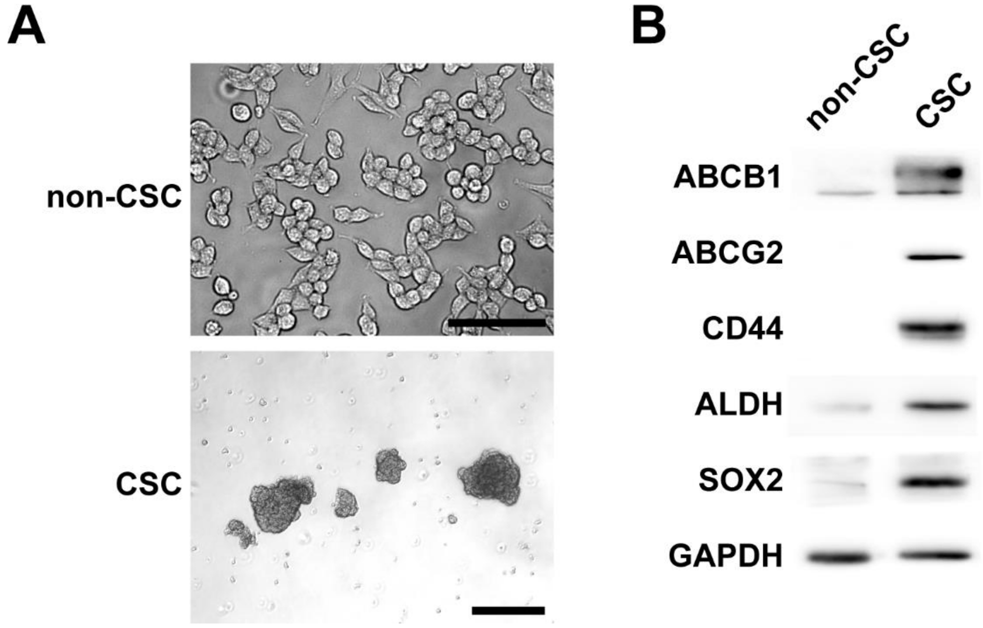

Figure S1. Increased expression of CSC markers in A2780-SP cells.
(A) Representative phase contrast images of A2780-SP and their parental A2780 cells. A2780 cells: scale bar, 100 µm; A2780-SP cells: scale bar, 500 µm. (B) Expression of CSC markers in A2780 and A2780-SP cells. The protein levels of CSC markers (ABCB1, ABCG2, CD44, ALDH, SOX2) and GAPDH were determined by Western blotting. Non-CSC: A2780 cells; CSC: A2780-SP cells derived from A2780 cells; CSC: cancer stem cell.

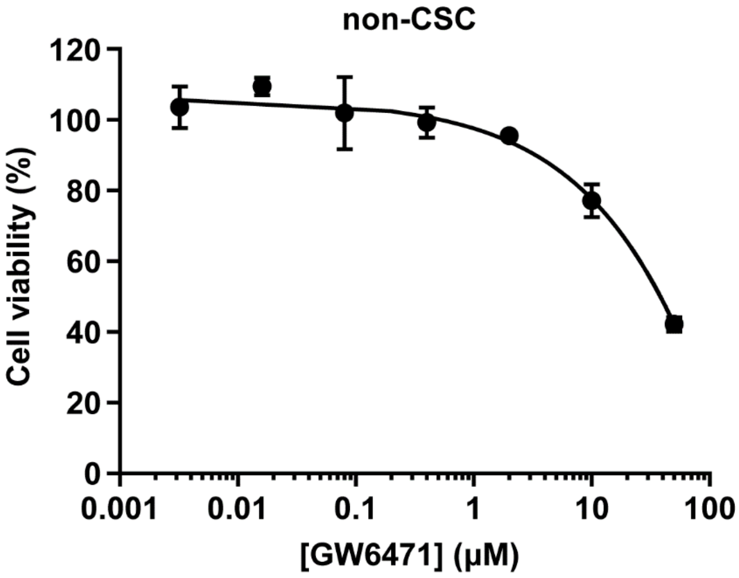

IC_{50} values of GW6471 on cell viability of A2780 and A2780-SP cells

	**non-CSC**	**CSC**
IC_{50}	35.7	17.2
95% confidence interval	25.1 – 55.4	14.6 – 20.6

Figure S2. Dose-dependent effects of GW6471 on cell viability of A2780 cells. A2780 cells (non-CSC) were treated with increasing doses of GW6471 for 48 h, and cell viability was measured using MTT assay. Data are presented as mean ± SEM. (n = 3). The IC_{50} values of GW6471 on cell viability of A2780 and A2780-SP (CSC) cells.

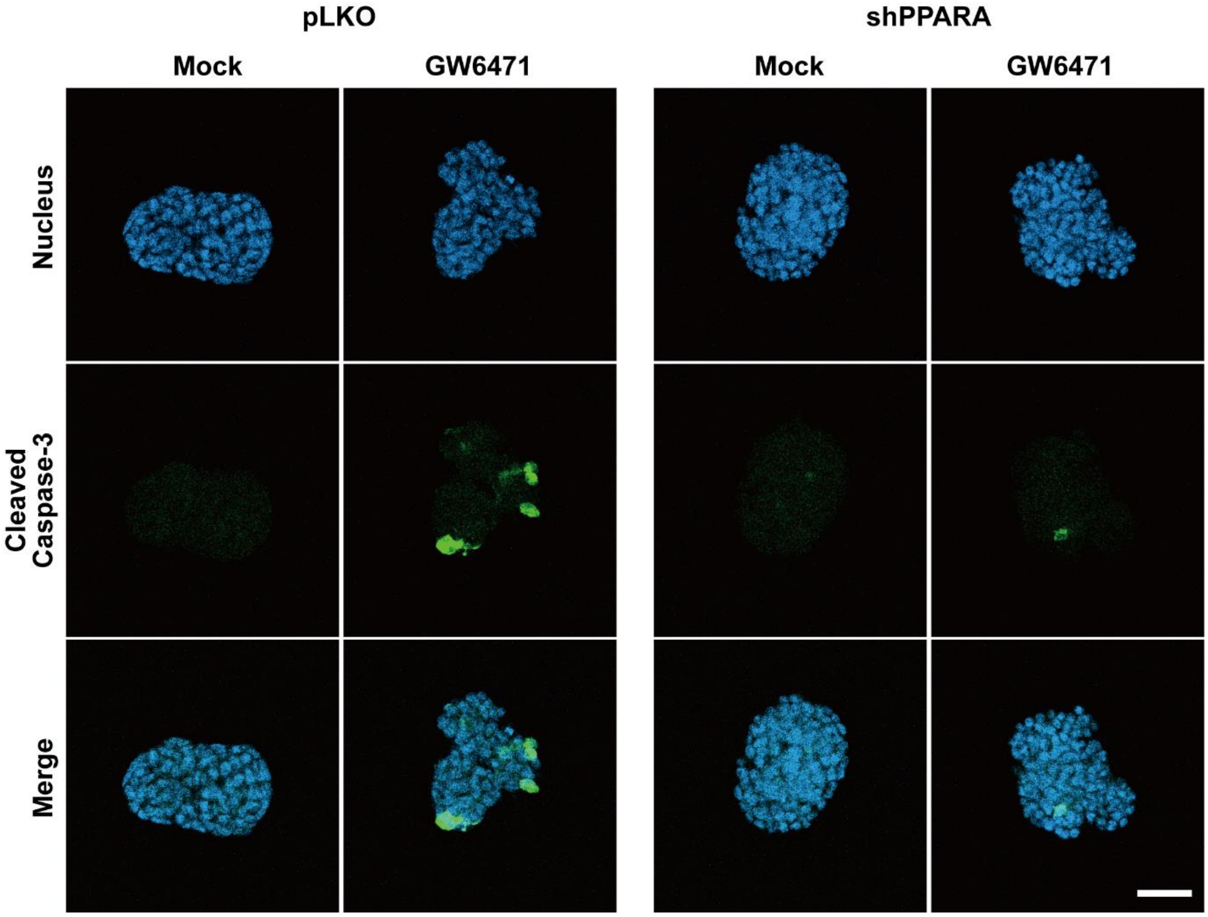

Figure S3. Effects of PPARα knockdown and GW6471 treatment on apoptosis in ovarian CSCs. Representative immunocytochemical images for the detection of cleaved caspase-3. A2780-SP cells, which were infected with lentivirus bearing control shRNA (sh-control) or PPARα shRNA (sh-PPARα), were treated with 0.1% DMSO (Mock) or 10 μM GW6471 for 48 h. The cells were immunostained with anti-cleaved caspase-3 antibody (green color) and counterstained with DAPI for nuclei staining (blue color), and merged images are shown. Scale bar, 50 μm. PPAR: peroxisome-proliferator-activated receptor; CSC: cancer stem cell; DMSO: dimethyl sulfoxide.

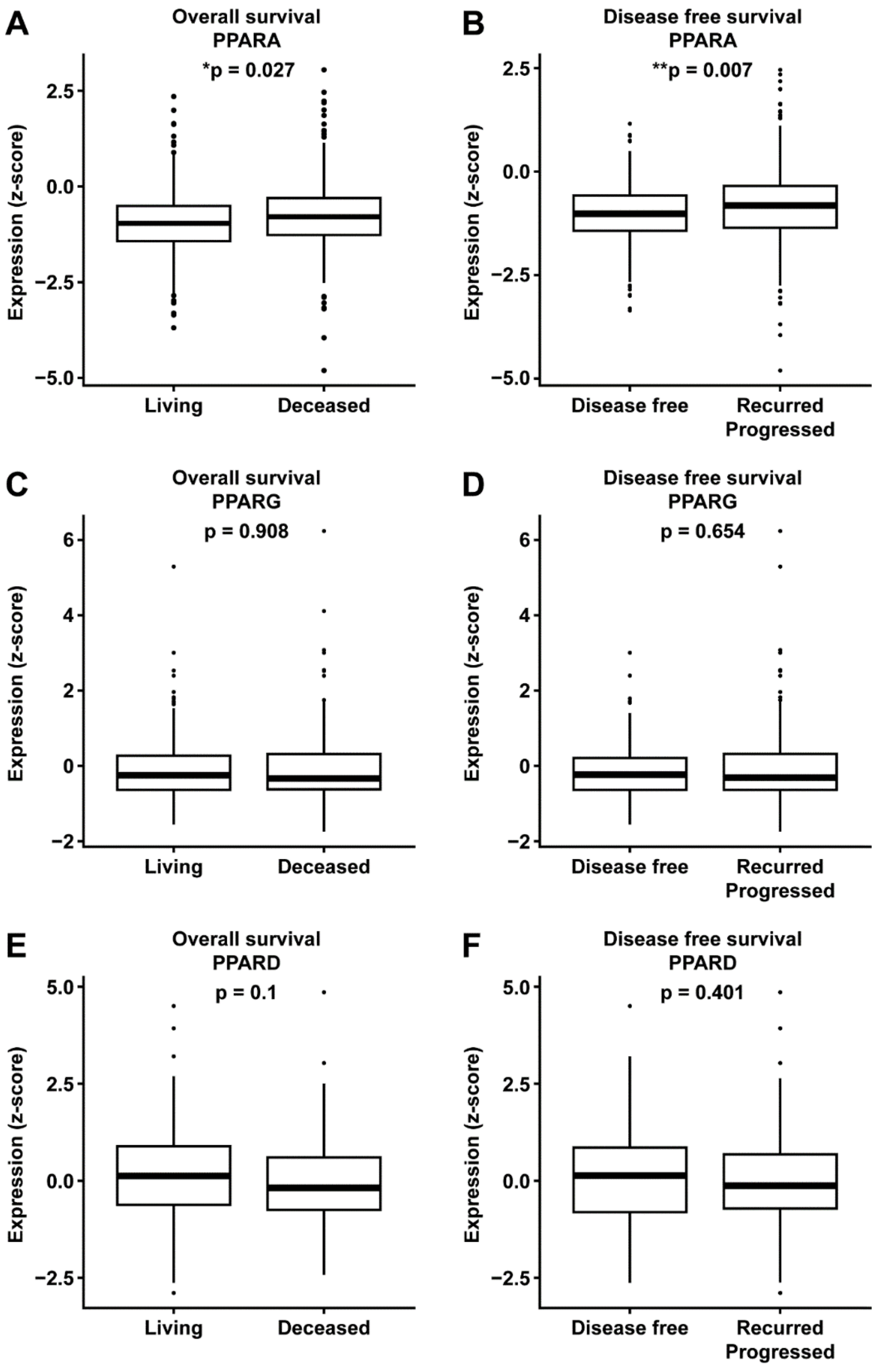

Figure S4. Comparison of PPAR subtype expression levels between groups stratified by clinical outcome. (A, C, E) Comparison of *PPARA*, *PPARG*, and *PPARD* expression levels, respectively, between patients who are alive (living) and patients who have died (deceased). (B, D, F) Comparison of *PPARA*, *PPARG*, and *PPARD* expression levels, respectively, between patients who are disease-free (disease-free) and patients who have experienced disease recurrence or progression (recurred/progressed). $^{*}p < 0.05$; $^{***}p < 0.001$. PPAR: peroxisome-proliferator-activated receptor.

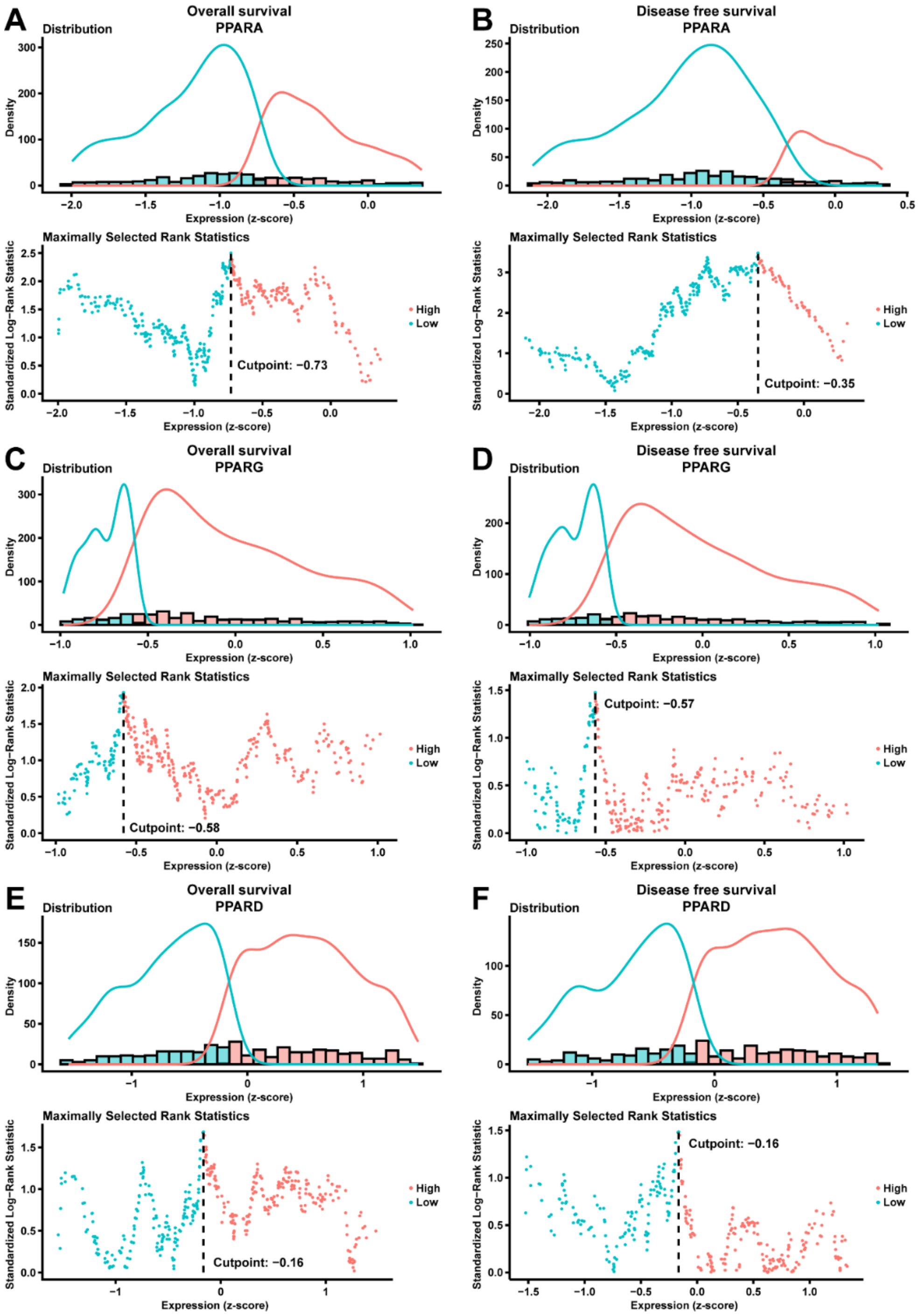

Figure S5. Determination of optimal cutoff values for *PPARA*, *PPARG*, and *PPARD* gene expression using maximally selected rank statistics. The vertical dotted line indicates the optimal cutoff value for each gene (i.e., the point of maximal statistical significance). PPAR: peroxisome-proliferator-activated receptor.
